# Transitional Care in Pediatric Brain Tumor Patients: A Systematic Literature Review

**DOI:** 10.3390/children9040501

**Published:** 2022-04-02

**Authors:** Florian Ebel, Ladina Greuter, Raphael Guzman, Jehuda Soleman

**Affiliations:** 1 Department of Neurosurgery, University Hospital of Basel, 4031 Basel, Switzerland; ladinaaurea.greuter@gmail.com (L.G.); raphael.guzman@usb.ch (R.G.); jehuda.soleman@gmail.com (J.S.); 2 Department of Pediatric Neurosurgery, University Children’s Hospital of Basel, 4056 Basel, Switzerland; 3 Faculty of Medicine, University of Basel, 4056 Basel, Switzerland

**Keywords:** pediatric brain tumor, neurofibromatosis type 1, tuberous sclerosis complex, transitional care, pediatric neurosurgery, pediatric neurology

## Abstract

Background: Due to advances in the treatment of pediatric brain tumors (PBT), an increasing number of patients are experiencing the transition from the pediatric to the adult health care system. This requires efficient transitional models. Methods: We systematically reviewed the literature regarding PBT concerning different transitional models and aspects of the transitional period. For this purpose, PubMed, Medline, and Embase databases were searched systematically through January 2022. Results: We reviewed a total of 304 studies, of which 15 were ultimately included. We identified five transition models described within the literature, while the most frequently mentioned ones were the “adult caregiver model” (45.5%), “joint caregiver model” (45.5%), “continued caregiver model” (27.3%), and the “specialized clinic model” (27.3%). During the transition, the most frequent challenges mentioned by the patients were the lack of knowledge about the disease by the adult health care professionals (62.5%) and the difficulty of establishing a new relationship with the new physician, environment, or hospital (37.5%). Conclusions: An efficient transitional model is mandatory for patients with PBT. Continuity in the treatment and care of the patient and their family is essential. For this purpose, in patients with PBT, the “continued caregiver model”, and for NF1 and TSC patients, the “specialized clinic model” seems optimal to offer continuity of care. If such models are unavailable, efficient communication with patients, families, and specialists in a multidisciplinary network is even more critical.

## 1. Introduction

Primary central nervous system (CNS) tumors are the second most common malignancy in the pediatric population [1]. Several advances and innovations in the treatment of pediatric brain tumors (PBT) have fortunately significantly increased the survival rate of these patients in recent decades [2].

However, increased survival also presents new challenges, such as transitioning from pediatric to adult health care systems. In addition, since long-term PBT survivors often suffer from multiple physical, cognitive, and neuropsychological sequelae of treatment, continuous health care is essential for these patients to reduce emotional stress, morbidity, and mortality in the long term [3,4,5,6]. In addition, PBT patients, at times, suffer from tumor-predisposing syndromes such as neurofibromatosis type 1 (NF1) and tuberous sclerosis complex (TSC), carrying a significant burden of chronic disease, aggravating treatment, and reducing the quality of life of these children.

Effective and efficient transition programs are needed to address these needs and ease the care of PBT patients and their families [7]. This review aims to provide a systematic overview of the existing literature on different transition models and the challenges of this transitional process in PBT patients.

## 2. Materials and Methods

The systematic literature search was carried out following the PRISMA Guidelines. We used a search term with the keywords “pediatrics, child, adolescent”, “brain tumor, CNS tumor, tuberous sclerosis, neurofibromatosis”, and “transition, transitional care” in the Pubmed/Medline and Embase databases with restrictions to English language, case series, case reports, clinical trials, controlled clinical trials, meta-analyses, randomized controlled trials, reviews, and systematic reviews. The detailed search term is provided in Supplementary Figure S1. We included all articles providing information on managing patients with PBT, including patients suffering from tumor-associated diseases, specifically TSC and NF1 during the transitional period. Two authors (F.E. and L.G.) independently assessed all results for eligibility. Where consensus opinion could not be reached, a third researcher (J.S.) decided whether to include the study or not. Studies that did not report information on transitional care in patients with PBT were excluded. The primary outcome measure was the different transitional model types described within the literature. Secondary outcome measures were difficulties and challenges occurring during the transitional period, the recommended age for transition, and described follow-up regimens for PBT patients when reaching adulthood.

In the analysis of the included reports, we named and summarized the different transition models described within the literature. Further, we summarized the most frequently described (by the care providers and the patients/caregivers) difficulties and challenges during the transition period. We summarized the recommended age for transition and follow-up time. Thereafter, the same variables were described for each group of patients (PBT, NF1, TSC), while the suggested follow-up diagnostics, based on the included reports, are described as well. The data presented is descriptive since most reports included are descriptive case studies or surveys.

## 3. Results

**Figure 1:**
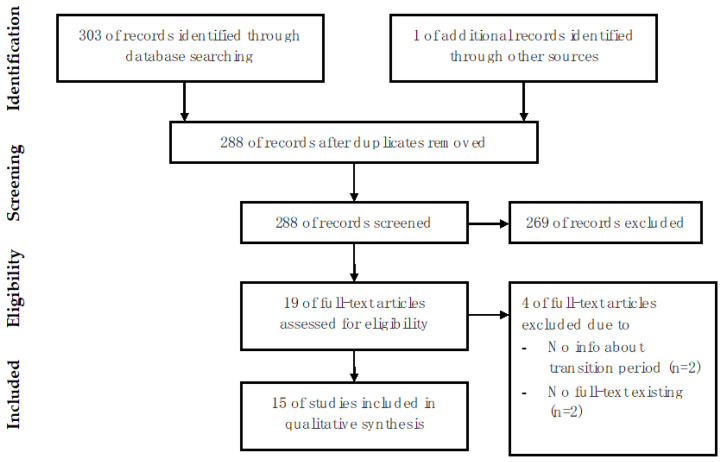
Selection of articles included in this review.

The systematic literature search yielded 304 results. After excluding 16 duplicates, we reviewed 288 articles and excluded 269 based on their title or abstract. Of the 19 full texts reviewed, four were excluded (lack of information on the transition period in two and no full text in two), whereon 15 studies were included in this systematic review [8,9,10,11,12,13,14,15,16,17,18,19,20,21,22] (Figure 1). The included studies consisted of eight reviews [11,12,15,16,17,18,19,21], four interviews/surveys [8,9,20,22], and three case series [10,13,14]. Eight studies discussed PBT patients [10,13,14,15,16,18,19,20], three emphasized on NF1 patients [12,17,22], and four on TSC patients [8,9,11,21].

### 3.1. Transition Models

Of the 15 studies included, 11 (73.3%) described different models for the transition period [8,9,10,12,13,15,16,17,20,21,22]. Overall, five models for the transition were described (Table 1):

- The “adult caregiver model”.
- The “shared caregiver model”.
- The “joint caregiver model”.
- The “continued caregiver model”.
- The “specialized clinic model”.

The characteristics of the different transition models are presented in Table 1. Of the 11 studies discussing transition models, five (45.5%) studies mentioned the “adult caregiver model”, two (18.2%) the “shared caregiver model”, five (45.5%) the “joint caregiver model”, three (27.3%) the “continued caregiver model”, and three (27.3%) the “specialized clinic model” (Table 1).

### 3.2. Difficulties and Challenges from the Patient’s Perspective during the Transition Period, Transition Age, Follow-Up Duration

Of the 15 included studies, eight (53%) describe difficulties and challenges from the patient’s perspective during the transition period [8,9,11,13,14,16,20,22]. The most frequent difficulty, the lack of knowledge of the adult health care professionals about the disease, was mentioned in five reports (62.5%) [8,9,11,13,16]. In three reports (37.5%), the difficulty of establishing a new relationship with the new physician, environment, or hospital was reported [8,16,20]. In two studies (25%), interruption of health care and the lack of communication about the upcoming transition were mentioned as problematic aspects (Table 2) [9,20,22]. Five studies (33%) discuss the recommended transition age, ranging from 14 to 21 years of age (Table 2) [8,10,12,21,22]. In two studies [10,22], the transition was undertaken at the age of 18, while in two studies between the ages of 16 and 21 years [8,12], and one study at the age of 14 [21]. Three studies recommend lifelong follow-up [12,15,16]. Two studies reported that patients with PBT had follow-ups for 11.2 and 14.8 years, respectively [13,14] (Table 2).

### 3.3. Pediatric Brain Tumor Patients

All eight studies, including PBT patients, describe a need for a transitional care model to accompany these patients into adulthood [10,13,14,15,16,18,19,20]. Of the total eight studies that addressed PBT, three (37.5%) mentioned the “continued caregiver model”, two (25%) mentioned the “shared caregiver model”, the “joint caregiver model”, and the “adult caregiver model”, respectively (Table 1). During the transitional period, the lack of specific knowledge of the adult health care professionals about the specific disease was described by 25% of the reports as the main problem in transition, while the development of new relationships with the adult professionals was described as the biggest hurdle in 25% of the reports (Table 2) [13,16,20]. The transition age is recommended by one study starting at the age of 18 years [10]. Two studies (25%) recommended lifetime follow-up [15,16]. Annual clinical follow-up was recommended by two studies (25%), and one study (12.5%) additionally recommended annual neuropsychological assessment (Table 2) [18,19,20].

Four (50%) reports described the medical problems faced by pediatric brain tumor patients [13,14,16,19]. All of them mentioned endocrinological, as well as neurocognitive late effects. Three out of four studies (75%) described secondary malignancies induced by radiation or chemotherapy and neurologic deficits [13,16,19]. Furthermore, psychosocial and cardiovascular disorders and hydrocephalus were described as possible medical problems during the follow-up [16,19].

### 3.4. Neurofibromatosis Type 1 Patients

All three studies focusing on NF1 patients reported on transition models, of which two (66%) mentioned the “specialized clinic model” and one (33%) the “joint caregiver model”. One study (33%) discussed the challenges during the transition period, highlighting the lack of communication regarding the upcoming transition, the lack of organization of subsequent follow-up visits, and the lack of a referral network to NF1-specialized physicians [22]. One study recommends a transition age between 16 and 20 years, and another study at 18 years [12,22]. Lifelong follow-up is recommended by one report [12]. The included studies looking at NF1 patients emphasize the importance of multidisciplinary follow-up [12,17,22]. According to two reports (66%), an annual clinical check-up, including height and weight measurement, skin examination, neurological examination, and blood pressure control, is recommended at follow-up [12,17] (Table 2).

### 3.5. Tuberous Sclerosis Complex Patients

All four studies focused on transitional management for TSC patients emphasized the importance of efficient transitional models [8,9,11,21]. Three of the four studies (75%) described the “adult caregiver model” [8,9,21]. Two (50%) studies described the “joint caregiver model” and one (25%) the “specialized clinic model” [9,21]. During the transitional period, the most frequently reported problem was a lack of knowledge about the disease by the adult health care professionals (75%) (Table 2) [8,9,11]. One study suggests a transition age of 16.5–21 years, or as early as 14 years [8,21]. None of the included studies reported the recommended follow-up duration. Overall, 75% of the studies recommended follow-up diagnostics regarding TSC. Two studies (50%) recommended annual or biannual neurological follow-up to control epilepsy and annual follow-up of neuropsychiatric status [8,9,21] (Table 2). In addition, one study suggests regular multidisciplinary consultations with MRI of the skull, EEG, renal function tests, and ultrasound imaging [8].

## 4. Discussion

Overall, the literature on transitional care in pediatric brain tumor patients is scarce. However, our systematic review showed that transitional care is of great importance for the patients’ and their families’ well-being and, thus, might also improve their quality of life. Therefore, a large population could potentially benefit from well-organized transitional programs. Hence, efficient models for this process are urgently needed.

Our systematic review provides an overview of the different transition models and how often they have been described. When summarizing the available literature, five different transition models are described. The two most frequently mentioned (45.5% each) models are the “adult caregiver model” and the “joint caregiver model”. Furthermore, we could show that the lack of expertise of the adult health care professionals in the respective (pediatric) disease was by far the difficulty most frequently mentioned by the patients and their families during the transitional period. The follow-up duration remains controversial, while recommendations based on the available literature cannot be made. Which examinations should be performed during follow-up and at what frequency depends mainly on the underlying lesion and/or disease (Table 2) and are described very heterogeneously amongst the different reports.

### 4.1. Transition Models

The most common transition models mentioned in the literature are the “adult caregiver model”, where a transfer from multidisciplinary pediatric health care to an adult primary care provider takes place, and the “joint caregiver model”, where temporary consultations are held with pediatric as well as adult physicians, such as oncologists or neurosurgeons, to facilitate a smooth transfer [8,9,10,12,15,16,21]. The “adult caregiver model” seems the most “natural” way of managing the transition period, since once a child or teenager becomes an adult, they are cared for by adult caregivers. However, as seen by the patients’ and families’ responses, this often leads to suboptimal treatment since a pediatric disease (such as PBT, NF1, TSC) is carried on to adulthood and does not become a disease of adult patients. Therefore, it is not surprising that the most common difficulty described by the patients was the lack of expertise and knowledge of the adult health care professionals. Therefore, one of the other four presented models seems beneficial for the transition of these patients since, in all, a pediatric caregiver with more knowledge and expertise in children’s diseases is at least involved in the patients’ future care. The decision regarding which of these four models (“joint caregiver”, “shared caregiver”, “continuous caregiver”, or “specialized clinics”) depends mainly on the given infrastructure of each institute and country. An example of the “joint caregiver model” is presented in the case series by Roux et al., describing 14 patients with pediatric brain tumors [10]. They used a “2-step process” to transfer from pediatric to adult health care, in which first the adult neurosurgeon was informed of the transfer by the pediatric neurosurgeon by referral letters and transfer of medical records. Subsequently, combined consultations with the pediatric and adult neurosurgeons were followed by consultations with the adult neurosurgeon only, who finally took over the treatment. This approach can surely provide a smoother transition from childhood to adulthood; however, a certain lack of knowledge remains once the pediatric neurosurgeon is no longer involved in the care. This can be overcome because the adult neurosurgeon is familiar with the primary treating pediatric neurosurgeon and can contact them or the team of pediatric caregivers at any time. An additional drawback of such a model is the joint presence of both disciplines leading to more financial and administrative effort and is more time-consuming for both physicians.

The “shared caregiver model” was only mentioned in two studies [15,16]. Since different disciplines share treatment in this model, there is some similarity to the “joint caregiver model”. Shared care allows for patient-focused long-term care, and in the study by Ellenbogen et al., high (88%) patient satisfaction was reported concerning this model [15].

The “continued caregiver model”, in which the attending physician, e.g., a pediatric neurosurgeon or neurologist, continues treatment into adulthood, was mentioned by three studies [13,15,20]. The clear advantages of this model are that, in principle, no transfer takes place, which provides full continuity in care, and the patient and their relatives are not stressed by a change in doctor or hospital. In addition, the risk of loss of information due to such a change is minimized. However, this model requires the appropriate infrastructure, which would involve a pediatric neurosurgeon or neurologist, who works part-time, or is involved within the counterpart adult department. Moreover, since an adult patient cannot be treated at a children’s hospital, the pediatric specialist would need a good network of adult specialists to treat complications or disease manifestation during adulthood.

Specifically, for NF1 and TSC, a total of three studies in the literature mentioned the “specialized clinic model”. Within this setting, patients receive lifelong care in a clinic specialized for these specific diseases [17,21,22]. This model of care is ideal, especially for such complex and rare diseases, such as NF1 and TSC, and PBT, which are often treated very differently than their adult counterparts [23,24]. The care and treatment of patients with these diseases requires a lot of experience and is ideally centralized in one place and managed by a multidisciplinary team of experts throughout adulthood. However, highly specialized centers are severely limited, as their establishment is only possible in tertiary care facilities and is usually associated with a significant financial investment. Therefore, for many patients and in many countries, such a model could be hard to establish and make accessible for patients.

Especially in the transitional models, where there is a change in the treating physician or several physicians are involved in the treatment simultaneously, the presence of good summary documentation can be essential for a smooth transition. Detailed documentation can prevent ambiguities and reduce the likelihood of misunderstandings. In addition, internal guidelines in the sense of standard operating procedures on how the transitional process should proceed could be supportive for the treating physicians and improve patient transition care. The above classification into five transitional models is based on the included literature and may reflect a simplification, as certain models can be used overlapping in the form of “hybrid models”.

### 4.2. Our Institution’s Transition Model

The children’s hospital in our center is directly adjacent to the adult hospital. Three fellowship-trained pediatric neurosurgeons are taking care of all pediatric cases. In addition, they are also affiliated in the adult neurosurgical department and are therefore familiar with the infrastructure of the children’s and the adult hospital. Therefore, at our institution we follow the “continuous caregiver model”. This offers the advantage that the pediatric neurosurgeons continue following PBT survivors during adulthood within the scope of their “adult” clinics. This leads to an ideal and continuous follow-up and care of these patients. The patients continue treatment and are, if needed, operated on in the future by the same neurosurgeon they and their family have known since childhood. Further, in case of recurrence, the patients’ case is still being discussed within the scope of pediatric tumor boards since the pediatric neurosurgeons have access to and are very much involved in these weekly discussions. This is of utmost importance since PBT, which recur in adulthood, still should be considered in most cases as PBTs and should be discussed from a pediatric oncological point of view rather than from an adult oncological point of view. Colleagues at McMaster Children’s Hospital (Hamilton, Canada) describe a similar model, where pediatric neuro-oncologists have primary responsibility, even in adulthood. If there is a recurrence, the patient is operated on by pediatric or adult neurosurgeons and then, after recovery, is followed up again by the pediatric neuro-oncologists [15].

NF1 or TSC patients are treated in our center according to the “specialized center model”. At our children’s hospital, these patients (children and adults) are cared for at the “center of neurocutaneous diseases”, where a multidisciplinary team of pediatric neurologists, pediatric neurosurgeons, neuro-ophthalmologists, pediatric neuroradiologists, and geneticists are involved in the care. Naturally, such a setup requires a network of specialists and, at times, good collaboration with the adult caregivers since invasive treatments are still undertaken in the adult hospital. Nevertheless, based on our experience and the results of this systematic review, the continuity and consistency of such a model are highly valued by the patients and their family members [20].

### 4.3. Difficulties and Challenges during Transition

Various problems can occur during the transitional period. The most common point mentioned by the patients and their families in the literature is that the adult health care professional lacks knowledge regarding the specific pediatric disease [8,9,11,13,16]. The second most common problem reported by patients and relatives was difficulty establishing a new relationship with the new physician [8,16,20]. Other problematic issues were that regular follow-up was often not scheduled after transition [22], insufficient information and communication about the disease was provided [20], and that often, there were still unresolved medical issues during the transition [14]. Based on these reports, it seems that patients and their families would prefer and benefit most from a “specialized clinic model” or a “continued caregiver model”.

### 4.4. Transition Age

Overall, data regarding optimal transition age in neurosurgery are very scarce. In the literature, the recommended age of transition for NF1 is between 16 and 20 years and TSC between 16.5 and 21 years [8,12,22]. One study suggests a transition age starting at 14 years, which seems relatively early since, to our knowledge, most adult hospitals care the earliest for patients above the age of 16 years [21]. If a model is practiced where the transition takes place, most reports recommend starting the transition process slowly, about 1–2 years before the actual transition, to allow the patient to get used to the idea of a doctor and/or hospital change and provide enough time to clarify uncertainties and questions in advance [8].

### 4.5. Follow-Up Duration

Basically, a very long or lifelong follow-up is recommended for the mentioned pathologies since long-term disease manifestations may occur [12,15,16].

Specifically, for patients with PBT, permanent medical surveillance with regular follow-up is recommended to manage late side effects of treatment, detect recurrences early, or assist in other medical and rehabilitative issues, to improve long-term survival and quality of life [3]. Several studies highlighted that the potential long-term effects of treatment and disease were numerous and included neurocognitive and neurological impairments, endocrinopathies, psychosocial problems, and secondary malignancies [3,16,25]. For example, a study by Vinchon et al. showed that 82% of PBT survivors suffered from late effects of the therapy or tumor recurrence after a median follow-up of 14.8 years, emphasizing the importance of long-term follow-up. Most frequently, endocrine (44%) or cognitive (43%) late effects occurred. Twenty-three percent of these patients had a progression of the initial tumor, and 14% developed new tumors during the follow-up [14]. These data illustrate and highlight the importance of sustained, long-term follow-up with medical connection and social support throughout adulthood.

Our follow-up protocol for PBT patients during adulthood is usually over many years, while when imaging has remained unremarkable and stable over at least 10 years, we conclude clinical follow-up only every 5–10 years. Naturally, the follow-up regimen is adapted to the patient’s specific histological and molecular diagnosis or suspected diagnosis (e.g., in the case of untreated low-grade gliomas) and the radiological results throughout follow-up. Therefore, a general recommendation for the follow-up duration cannot be given, and it is of utmost importance to discuss these patients within the scope of (pediatric) neurooncological conferences throughout adulthood.

### 4.6. Follow-Up Diagnostics

For the follow-up regime in patients with PBT, the type of tumor and the extent of resection influence which follow-up protocol and imaging intervals are performed. For example, high-grade or partially resected tumors require more frequent follow-up than low-grade or completely resected tumors [26,27]. Regarding long-term follow-up, guidelines by the children’s oncology group exist [28]. To the best of our knowledge, there are no prospective studies regarding different surveillance protocols in PBT.

For patients with neurocutaneous diseases such as NF1 and TSC, based on our systematic literature review, no clear recommendation can be made regarding the type of follow-up examinations and their frequency, as this was not the aim of our work. However, there are guidelines, such as the French national guidelines regarding NF1 and the 2012 International Tuberous Sclerosis Complex Consensus Conference guidelines regarding TSC, which recommend regular clinical and radiological controls, some of which were also mentioned within the studies we included (Table 2) [29,30].

### 4.7. Future Directions of Transitional Care in Patients with PBT

Preferably, transitional care should be undertaken within the scope of specialized transitional health care centers. Within these centers, ideally trained pediatric and adult health care specialists provide long-term interdisciplinary care of these patients. In addition, the staff would have specialized training in dealing with patients and relatives in the transitional period and would optimally cover their needs.

Furthermore, we believe that more studies are necessary to investigate the needs of patients and their relatives in the challenging transitional period. This could be completed, for example, by collecting patient-reported outcome measures. Moreover, it seems that there is not yet sufficient awareness within the affected communities (adult and pediatric oncology, neurology, and neurosurgery) on the importance of careful and efficient transitional care in patients with PBT. Interdisciplinary congresses or courses between pediatric and adult health care specialists might be helpful in order to increase the awareness for an effective transitional period and its challenges and help minimize the gap between the adults and pediatric health care providers. Finally, more studies with long-term data from patients with PBT, describing the risk of recurrence, rates of malignant transformation, as well as the cognitive and social outcomes that occur during and after the transitional period, are needed. Adult health care providers can be better prepared to care for such complex patients when provided with these findings.

### 4.8. Limitations

This systematic review has several limitations. First, only two databases, namely PubMed and Embase, were searched, and we included only reports in English. This may have led to the omission of important studies. Second, besides PBT, NF1, and TSC, other diseases, such as Sturge Weber and VACTERL association, were not included in our analysis, although the transition is an issue also in these patients. However, since these syndromes usually do not encounter PBTs, they were excluded from this analysis. Furthermore, other medical disciplines are often confronted with transitional care. Indeed, there is literature in these fields about transitional care, but within the focus of this study, which was set primarily on neurosurgical patients with PBT, NF1, and TSC, these papers were not considered. Third, due to the heterogeneous and scarce data, no definitive recommendation can be made on the choice of transition model or follow-up diagnostics. Fourth, due to the lack of data, no analysis of the appropriateness of the transitional models based on age and diagnosis could be made. Fifth, based on the included literature, the distinction between the five transitional models we mentioned might be a simplification, since some models can be overlapping leading to a “hybrid” of the mentioned models.

Nevertheless, to the best of our knowledge, this is the first systematic literature review on the different transition models in patients with PBT and their challenges.

## 5. Conclusions

An efficient transitional model is essential for high-quality medical care of PBT patients during adulthood. Patients and relatives often described this as an essential factor for a good transition experience. The most often described model is the “adult caregiver model”, however, based on the experience described by caregivers and reports by the patients and their families, this model seems insufficient. For PBT, the “continued caregiver model” and the “specialized clinic model” for neurocutaneous diseases seem appropriate and ideal. Recommended guidelines on transitional models, the follow-up time and regime, and the ideal age of transition cannot be given due to the paucity of the literature and the lack of consensus amongst reports.

## Figures and Tables

**Table 1:** Overview of different transitional models and their advantages and disadvantages.

Models	Description	Advantages	Disadvantages	Mentioned in (%)	Subcategory (n)
Adult caregiver model	Transition from multidisciplinary pediatric care to an adult primary care provider	Everything is coordinated and organized by a primary care provider. This gives the care provider a good overview and reduces the risk of information being lost. (A)	Lack of knowledge about the specific disease from the adult care provider. (A)	5/11 studies (45.5%) [8,9,15,16,21]	PBT (2) TSC (3)
Shared caregiver model	Shared care by two or more providers of different specialties (e.g., GP and cancer center)	Patient-focused long-term follow-up [15] 88% patient satisfaction [15]	Reduced resources available at each visit [15]	2/11 studies (18.2%) [15,16]	PBT (2)
Joint caregiver model	Joint consultations with the previous and future physician for a certain period during the transition period	Opportunity to introduce the new adult specialist [10,12] Minimize the loss to follow-up [9] A pediatric physician is available to support and educate the adult team [21]	Requires time from both parties [21] High administrative effort [21]	5/11 studies (45.5%) [9,10,12,16,21]	PBT (2) NF1 (1) TSC (2)
Continued caregiver model	Continued follow-up by the pediatric specialist team (e.g., pediatric neurology or neurooncology team)	Continuity in treatment by the same caregiver minimizes the risk of information loss (A) High comfort for the patient, as there is no change in routine (A)	Adult patients within the care of pediatricians (A) Care by multiple providers such as adult family doctors and pediatricians (A) In the case of a recurrence, a pathway for surgical management would need to be defined [15]	3/11 studies (27.3%) [13,15,20]	PBT (3)
Specialized clinic model	Patients are followed up and treated lifelong in a specialized clinic (e.g., neurocutaneous disease clinic) treating pediatric and adult patients	All physicians have access to both the pediatric and adult electronic medical records [21] The same specialists, who have developed expertise in this condition, often follow the patients at all ages [21] Continuity of care [21]	Limited to a few locations. Not available in rural areas. (A) Requires extensive human and financial resources (A)	3/11 studies (27.3%) [17,21,22]	TSC (1) NF1 (2)

Abbreviations: GP: general practitioner; PBT: pediatric brain tumor; NF1: neurofibromatosis type 1; TSC: tuberous sclerosis complex; (A): authors’ comments.

**Table 2:** Overview of the problems, suggested age of transition, follow-up duration, and follow-up diagnostics based on the included studies.

	PBT Patients	NF1 Patients	TSC Patients
Problems during transition	Separation difficulties between the pediatric health care professional and the patient [16] Transfer to adult health care often resulted in discontinuation of care [20] Lack of communication about the upcoming transition to adult health care [20] Development of new relationships with adult professionals [16,20] Insufficient information about the disease and treatment [20] Lack of specific knowledge about the disease by the adult health care professionals [13,16] Unresolved medical issues at the beginning of the transitional process [14]	Lack of communication about the upcoming transition to adult health care [22] Lack of organization of regular follow-up [22] Lack of referral network to NF1-specialized physicians [22]	Lack of knowledge about the patients’ medical history [8] Lack of specific knowledge about the disease of the adult health care professionals [8,9,11] Health care often resulted in discontinuation of care [9] Loss of connection with the pediatrician who previously provided treatment and follow-up [9] Development of new relationships with adult professionals [8]
Suggested age of transition	From the age of 18 years (mean 19.6 y) [10]	16–20 years [12] 18 years [22]	14 years [21] 16.5–21 years [8]
Follow-up duration	Mean 11.2 years [13] Mean 14.8 years [14] Lifelong [15,16]	Lifelong [12]	N/A
Follow-up diagnostics *	Yearly clinical follow-up for reassurance [19,20] Yearly neuropsychological assessments [18]	Yearly clinical follow-up (including skin and neurologic exam and BP, height, and weight) [12,17] Ophthalmologic exam every 1–2 years [17] Single whole-body MRI at transition [17] Women: extra breast cancer screening [17]	Biannual or yearly neurologic follow-ups depending on control of epilepsy [8,21] Annually screening for TAND [8,9] Every 3–5 years ECG to monitor for conduction defects [21] Annually ophthalmologic evaluation [21] Every 1–3 years, renal function assessment and imaging [21] Annual blood pressure assessment [21]

* The follow-up diagnostics mentioned are based on the included studies and do not represent the comprehensive guidelines for follow-up. Abbreviations: PBT: pediatric brain tumor; NF1: neurofibromatosis type 1; TSC: tuberous sclerosis complex; TAND: tuberous sclerosis complex-associated neuropsychiatric disorders; ECG: electrocardiogram; BP: blood pressure; N/A: not applicable.

## Data Availability

The data presented in this study are available on request from the corresponding author.

## Supplementary Materials

The following supporting information can be downloaded at: https://www.mdpi.com/article/10.3390/children9040501/s1, Figure S1: Search strings in pubmed and embase.
