# Supplementary material for: Transitional Care in Pediatric Brain Tumor Patients: A Systematic Literature Review
Source: Children (Basel). 2022 Apr 2;9(4):501. doi: 10.3390/children9040501 (PMC9026288; doi:10.3390/children9040501)
Supplement: Supplementary file 1 [file children-09-00501-s001.zip › children-1613545-supplementary.pdf]

Supplementary:

PubMed

(("Pediatrics"[MeSH Terms] OR "Child"[MeSH Terms] OR "Adolescent"[MeSH Terms] OR "Pediatrics"[Title/Abstract] OR "Child"[Title/Abstract] OR "Adolescent"[Title/Abstract]) AND ((((((((((("Central Nervous System Neoplasms"[Mesh]) OR "Brain Neoplasms"[Mesh]) OR "Medulloblastoma"[Mesh]) OR "Glioma"[Mesh]) OR "Astrocytoma"[Mesh]) OR "Neuroectodermal Tumors, Primitive"[Mesh]) OR "Ependymoma"[Mesh]) OR "Craniopharyngioma"[Mesh]) OR "Neoplasms, Germ Cell and Embryonal"[Mesh]) OR "Meningioma"[Mesh]) OR "Tuberous Sclerosis"[Mesh]) OR "Neurofibromatoses"[Mesh])) AND ("Transition to Adult Care"[MeSH Terms] OR "transition"[Title/Abstract] OR "transitional care"[Title/Abstract])

Embase

(pediatrics:ab,ti OR child:ab,ti OR adolescent:ab,ti) AND ('brain tumor':ab,ti OR 'central nervous system tumor':ab,ti OR medulloblastoma:ab,ti OR glioma:ab,ti OR astrocytoma:ab,ti OR 'primitive neuroectodermal tumor':ab,ti OR ependymoma:ab,ti OR craniopharyngioma:ab,ti OR 'germ cell tumor':ab,ti OR meningioma:ab,ti OR 'tuberous sclerosis':ab,ti OR neurofibromatosis:ab,ti) AND ('transitional care':ab,ti OR 'transition to adult care':ab,ti OR transition:ab,ti)

Figure S1. Search strings in pubmed and embase.
